# An updated review of gastrointestinal toxicity induced by PD-1 inhibitors: from mechanisms to management

**DOI:** 10.3389/fimmu.2023.1190850

**Published:** 2023-06-19

**Authors:** Yiyu Cheng, Fangmei Ling, Junrong Li, Yidong Chen, Mingyang Xu, Shuang Li, Liangru Zhu

**Affiliations:** Division of Gastroenterology, Union Hospital, Tongji Medical College, Huazhong University of Science and Technology, Wuhan, China

**Keywords:** programmed cell death-1 (PD- 1), immune-related adverse effects (irAEs), colitis, mechanisms, rechallenge, microbiome, biomarkers, novel drug treatment

## Abstract

PD-1 inhibitors, as one of commonly used immune checkpoint inhibitors, enable T-cell activation and prevent immune escape by blocking the PD-1/PD-L1 signaling pathway. They have transformed the treatment landscape for cancer in recent years, due to the advantages of significantly prolonging patients’ survival and improving their life quality. However, the ensuing unpredictable immune-related adverse effects (irAEs) plague clinicians, such as colitis and even potentially fatal events like intestinal perforation and obstruction. Therefore, understanding the clinical manifestations and grading criteria, underlying mechanisms, available diverse therapies, accessible biomarkers, and basis for risk stratification is of great importance for the management. Current evidence suggests that irAEs may be a marker of clinical benefit to immunotherapy in patients, so whether to discontinue PD-1 inhibitors after the onset of irAEs and rechallenge after remission of irAEs requires further evaluation of potential risk-reward ratios as well as more data from large-scale prospective studies to fully validate. At the end, the rare gastrointestinal toxicity events caused by PD-1 inhibitors are also sorted out. This review provides a summary of available data on the gastrointestinal toxicity profile caused by PD-1 inhibitors, with the aim of raising clinicians’ awareness in daily practice, so that patients can safely benefit from therapy.

## 1 Introduction

Tumors are likely to be the dominant cause of death in the future (1). In response to an upward trend in morbidity and mortality, it is imperative to reduce the global cancer burden gradually and steadily. Compared with the toxicity of chemotherapy drugs, the limitation of surgery timing as well as targetable driver mutations of targeted therapy, immune checkpoint inhibitors (ICIs) stand out as an emerging immunotherapy approach to combat cancer by regulating the immune function of the organism and tumor microenvironment (TME). Programmed cell death-1 (PD-1) inhibitors as a type of ICIs are designed to boost T-cell activation and levels of proinflammatory cytokines along with concentrations of autoimmune antibodies against cancer by blocking PD-1 protein expression (2). Accordingly, it opens an avenue of tumor treatment and rapidly expanded to first-line settings for its powerful clinical efficacy on avoiding immune escape of tumor cells (3).

Despite ongoing progress in PD-1 monoclonal antibodies including nivolumab, pembrolizumab, and sintilimab, the disrupted balance of immune tolerance and systemic inflammatory reactions in a seemingly unpredictable fashion will result in organ-specific toxicities. Such immunogenic adverse events that occur during or after ICI therapies are described as immune-related adverse events (irAEs) (4).

Colon inflammation (colitis), with or without small bowel inflammation (enterocolitis), are the dominant adverse events associated with anti–PD-1 therapy, manifested as abdominal pain, diarrhea, blood, and mucus in stools (5). The incidence of diarrhea was reported to be 12.1–13.7%, and the incidence of colitis was 0.7–1.6% in patients with anti–PD-1 (6). In addition, an increasing number of rare and potentially life-threatening irAEs are being reported such as bowel perforation or obstruction. A meta-analysis involving 19,217 oncology patients demonstrated that fatal toxic effects induced by anti–PD-1 agents occur at a rate of 0.36%, with death from colitis accounting for approximately 0.066% (7). The median time to onset of gastrointestinal adverse events was about 40 days and, to ICIs-related, fatality was about 43 days after treatment (7, 8). Several patients opt for combined anti–PD-1 and anti-cytotoxic T lymphocyte antigen 4 (CTLA-4) to achieve a more satisfactory efficacy, but it is accompanied by more and faster occurrence of adverse events (9), of which pembrolizumab plus ipilimumab had the shortest median time to onset irAEs (10).

Unexpectedly, adverse events imply better outcomes. More extensive endoscopic inflammation rather than being limited to the left colon, acute histological inflammation, higher grade colitis, and recurrent diarrhea tend to have better long-term survival outcomes (11). There is no doubt that high-grade irAEs account for temporary or permanent discontinuation of immunotherapy. Up to 3–12% of patients forgo further anti–PD-1 therapy, although most irAEs can be ameliorated by symptomatic therapy with (or without) corticosteroids (6, 12). Given that risk and benefit may affect the patients’ life quality, the question of whether to restart ICI therapy after adverse events has become a dilemma for clinicians.

Immune cells and inflammatory factors are indispensable in the progression of irAEs, and even specific strains of gut microbes are mechanistically linked to susceptibility of irAEs. For instance, the phylum *Bacteroidetes* is associated with a lower incidence of immune-associated colitis, whereas the phylum *Firmicutes* is associated with a higher incidence of colitis (13). Despite continuous exploration in recent years, the pathogenesis of toxicity is not well clarified. The high prevalence has led to classification method and empirical management guidelines being introduced and updated. However, standardized irAEs guidelines are not adequate enough for lack of a wealth of experience and high-quality evidence. Consequently, it is crucial to focus on biomarkers, establish risk stratification models, and perform routine testing or biopsies if necessary (5, 14).

In this review, gastrointestinal side effects after anti–PD-1 therapy are in the spotlight. By comprehensively summarizing relevant mechanisms, enriched therapeutic arsenal, potential predictors and risk factors, we hope to provide a theoretical basis and shed light on novel therapeutic strategies in PD-1–mediated irAEs that address toxicity without eliminating antitumor efficacy, allowing patients to safely benefit from treatment.

## 2 Clinical manifestations and grading criteria

Diarrhea is a disorder characterized by an increase in frequency and/or loose or watery bowel movements. Due to intestinal inflammation, colitis often manifests as diarrhea, abdominal pain, distension, blood or mucus in the stool, fever and even upper gastrointestinal symptoms like nausea and vomiting.

Clinicians typically choose endoscopy with biopsy to assist in verification when they find suspected patients. Colitis has a range of presentations on endoscopy, including ulcerations or non-ulcerative inflammatory morphology such as diffuse or patchy erythema, inflammatory exudate, loss of vascular pattern, aphthae, edema, friability, erosions or granular mucosa. The histological features are generally similar to acute colitis, manifested as intestinal lamina propria expansion, intraepithelial neutrophilia and neutrophil crypt abscess; but may also present as chronic inflammation, described as basal lymphocytic infiltrate, cryptic architecture distortion, and Paneth cell metaplasia (11). Patients with severe colitis symptoms need to be alert for complications like bowel perforation and intestinal obstruction, and CT scan can be used to evaluate bowel wall thickening, colonic distension, mesenteric vessel engorgement, abscess, and perforation (2).

Immune-related toxicities were graded by based on Common Terminology Criteria for Adverse Events (CTCAE) v5.0. For details, check the table below. Grade 1 (G1) diarrhea is defined as an increase of less than four bowel movements per day as well as a mild increase in ostomy output compared with baseline. G1 colitis is often asymptomatic but detectable by imaging changes. G2 diarrhea is defined as an increase of 4-6 bowel movements per day, moderate increase in ostomy output over baseline, and limited instrumental activities of daily living (ADLs). Patients with G2 colitis suffer from mild or moderate abdominal pain and mucus or blood in stool. G3 diarrhea is defined as more than seven bowel movements per day, severe increase in ostomy output from baseline as well as limited self-care ADLs such as bathing, feeding and taking medications, and the involved patients usually require hospitalization. G3 colitis often presents with severe abdominal pain and peritoneal signs. G4 diarrhea/colitis may be combined with life-threatening consequences such as perforation, bleeding or toxic megacolon that require urgent intervention. All G5 diarrhea/colitis patient outcomes are defined as death (**Table 1**).

**Table 1:** Grading of irAEs referring to the CTCAE v5.0.

Gastrointestinal disorders	Grade 1	Grade 2	Grade 3	Grade 4	Grade 5
Diarrhea	Increase of less than 4 stools per day and mild increase in ostomy output compared with baseline.	Increase of 4–6 stools per day and moderate increase in ostomy output compared with baseline; limited instrumental ADLs.	Increase of more than 7 stools per day and severe increase in ostomy output compared with baseline; limited self-care ADL such as bathing, feeding and taking medications; hospitalization indicated.	Life-threatening consequences such as perforation, bleeding or toxic megacolon; urgent intervention indicated.	Death
Colitis	No clinical symptoms, but imaging changes may be present.	Mild or moderate abdominal pain as well as mucus or blood in stool.	Severe abdominal pain and peritoneal signs.	Life-threatening consequences such as perforation, bleeding or toxic megacolon; urgent intervention indicated.	Death

## 3 The underlying mechanisms

Most scholars are in favor of viewpoints that the occurrence of irAEs is regulated by a subclinical or latent autoimmune state and patients tend to develop the corresponding clinical symptoms after ICI treatment (15). In that case, what is the pathogenesis of irAEs actually? It is still under continuous investigation, but basically revolves around infiltration by T lymphocytes and innate lymphocytes, the cytokine storm, as well as B lymphocytes-mediated elevation of autoimmune antibody concentrations. Clarifying the immune pathways involved in irAEs will assist in the development of therapeutic modalities that can prevent or mitigate irAEs without compromising antitumor immunity.

### 3.1 Changes in immune cell profile

#### 3.1.1 Bulk T-cell receptor diversity

Immune cells such as T cells can express PD-1, and when PD-1 binds to PD-L1 ligands expressed by cytokine-stimulated tumor cells, it can aggregate with T-cell receptor (TCR) and induce the dephosphorylation of the proximal TCR signaling molecules, thereby inhibiting T-cell activation. PD-1 inhibitors are intended to enhance T-cell anti-tumor response by blocking the above process (13).

Andrews and colleagues evaluated the immune profile of patients’ peripheral blood and concluded that high-grade irAE was strongly tied to increased TCR diversity and enhanced T-cell expansion at baseline (16). Likewise, Jing et al. proposed that TCR diversity and CD8^+^ T-cell abundance showed the greatest correlation with irAE (17).

To further understand the mechanisms involved, Lozano et al. collected peripheral blood samples from metastatic melanoma patients treated with anti–PD-1 monotherapy or combination ICIs of anti–PD-1 and anti–CTLA-4, and built a strong connection between severe irAEs within 3 months of treatment initiation and pre-treatment circulating activated CD4^+^ memory T-cell abundance as well as bulk TCR diversity (15). In addition, a greater magnitude of TCR clonal expansion may contribute to more rapid progression of severe irAEs (15). Regrettably, the study lacks sufficient evidence for delayed irAEs beyond 3 months; in addition, it needs to determine whether the conclusions drawn in metastatic melanoma can be generalized to other tumor types by prospectively in-depth validation.

#### 3.1.2 The T helper 1-skewed phenotype

T-box expressed in T cells (T-bet) was identified as the master regulator for the differentiation of T helper 1 (Th1) cells. Its expression is significantly associated with prognosis, attributed to the promotion of Th1 cell differentiation. In human tumor tissues, when T-bet is lowly expressed, the Th1/Th2 balance is disrupted and tilted toward Th2 cells and tumor immune escape occurs; in contrast, high expression of T-bet promotes Th1 cell differentiation and exerts significant anti-tumor effects, which may predict a positive prognosis (18, 19).

After summarizing two cases of nivolumab-induced severe colitis, Yoshino et al. proposed that the mechanism of their adverse events had greater likelihood of a dominant response of Th1 cells, demonstrated as a strong infiltration of CD4^+^ cells expressing T-bet (20). When compared intestinal samples with irAEs colitis to healthy intestinal samples, Reschke et al. noticed an expansion of the Th1/Tc1-type cytokine profile of Tissue-resident memory T cells (T_RM_) in irAEs colitis, with increased expression of IL-15 required for their differentiation and survival (21). Markedly upregulated TNF-α; IFN-γ secreted by Th1/Tc1; and chemokines such as CXCL9, CXCL10, CXCL11, and dominant expression of checkpoint receptors (e.g., PD-1 and CTLA-4) were also detected. Interestingly, the adhesion molecule ITGA4, which is hardly expressed in healthy colon, was found a growing number in irAEs colon samples (21).

As a result, it is reasonable to speculate the Th1-skewed phenotype is of potential mechanisms driving the correlation between irAE and antitumor response

#### 3.1.3 Reactivation of effector T cells

Blocking the PD-1 signaling pathway with anti–PD-1 agents reactivates exhausted effector T cells to kill tumor cells, and the autoantigens released by tumor lysis contribute to autoimmunity. In addition, that is exactly what happened; the occurrence of irAEs was accompanied by high levels of CD4^+^ and CD8^+^ effector memory T cells (22). PD-1 inhibitors cause hyper-responsiveness cytotoxic T lymphocytes (CTLs) and Th1 cells in irAEs, with excessive production of interferon γ (IFN-γ), granzyme B (GZMB), interleukin 12 (IL-12), and tumor necrosis factor α (TNF-α) (23).

Tissue-resident memory T cells (T_RM_), a class of uncirculated lymphocytes persist in peripheral tissues, are the initial mainstays defending against local infections and play a key role in tumor immunotherapy and autoimmune diseases (24). By single-cell RNA sequencing (scRNA-seq) and flow cytometry, Luoma et al. (25) first analyzed immune cell changes at the single-cell level in intestinal biopsy samples from melanoma patients with ICI-induced colitis, revealing that cytotoxic T cells and proliferating T cells derived from T_RM_ were enriched in colitis samples, with high expression of IFN-γ and GZMB. Since T_RM_ is already abundant in healthy colon, colitis may be early irAEs attributed to the rapid activation of T_RM_ after recruitment of T cells from the blood.

#### 3.1.4 Regulatory T cells

Regulatory T cells (Tregs) modulate the formation of an immune-suppressive microenvironment and promote tumor immune evasion. Conflicting opinions exist in regards to the role Tregs play in ICI-induced colitis.

Anti-CTLA-4–mediated antitumor immunity and irAEs are attributed to Tregs depletion (23). Luoma and colleagues discovered that Treg cluster 1 cells were concentrated on colitis caused by anti–CTLA-4 monotherapy or in combination with anti–PD-1, expressing some genes specific to Th1 cells, such as IL-12 receptor and CXCR3, therefore revealing insufficient evidence of regulatory T-cell depletion in colitis (25). However, the role of Tregs in anti–PD-1 monotherapy-mediated colitis deserves more research to prove.

#### 3.1.5 Elevation level of innate lymphocytes

Innate lymphocytes (ILCs) are widely distributed in the human bodies, especially in intestinal mucosal tissues, and group 3 innate lymphoid cells (ILC3) are responsible for intestinal homeostasis by secreting IL-22, IL-17 and GM-CSF (26).

A growing body of clinical evidence suggests that some patients develop hyperprogressive disease (HPD) with accelerated tumor growth after anti-PD1 immunotherapy. Probing the mechanism revealed that the ILC3s are activated and upregulated in these patients, helping to suppress T cells responses and inducing T cells death, which hastens disease progression and leads to poor prognosis (27).

Wang et al. observed in tumor-bearing mice a correlation between the severity of immune-induced colitis and the increased number of ILC3s in their intestinal mucosa, regardless of ILC1 and ILC2; reduction of the mucosal number of ILC3s improved the colitis symptoms, and the inflammatory indicators such as IL-17 showed a tendency to decrease to normalization (28).

In general, perhaps as a result of T cell reactivation, inhibition of ILC3 reduces the risk of developing HPD after treatment, in parallel with reducing the development of ICI-related adverse effects such as colitis. As a matter of concern, however, it was proposed that a decrease in ILC3 may drive resistance to ICI therapy in colorectal cancer patients (29). More in-depth exploration of ILC3’s potential contribution to immunotherapy is warranted, but in any case, its powerful immunomodulatory properties hold great promise as a target for cancer therapy.

#### 3.1.6 B cells activation and autoantibodies

B-cell activation and subsequent autoantibody production play a considerable role in the development of irAEs. However, the role of B cells in antitumor immunity is still controversial.

The percentage and absolute number of B cells decreased with irAEs and the magnitude of the decrease was strongly positively correlated with the severity of the irAE, enabling early identification of patients at risk of toxicity through prophylactic monitoring of B cells (22, 30). A decline in circulating B cells and an increase in CD21lo B cells and plasmablasts are detectable in the first cycle after ICI treatment. Of note, patients with early alterations in the B cell populations were more likely to develop grade 3 or higher irAEs by the 6th month of ICI use, with these changes preceding toxicity by a median of 3 weeks (31).

CXCL13, known as B-lymphocyte chemokine ligand, regulates the homing of B cells to lymphoid follicles by binding to the receptor CXCR5. Anti-PD-1–induced irAEs were followed by upregulated plasma expression of CXCL13 (32). Another team observed that irAEs lead to the accumulation and dysfunction of CXCR5^+^ invigorated T follicular helper cells (Tfhs) in the germinal centers, ultimately leading to B cell-mediated autoantibody overproduction (23).

Removal of B cells from the mice revealed a decrease in circulating immunoglobulin G (IgG) levels, which were previously elevated, as well as alleviated organ tissue damage (32). Anti–PD-1 therapy triggers B-cell–mediated antibody-dependent irAEs and IgG levels in baseline serum may predict the development of irAE (33). More than that, one prospective study evaluated the association of baseline serum IgG and its subclasses levels with antitumor response and survival after ICI therapy in 49 patients, revealing that high-rise levels of total IgG (> 9.66 g/liter, *P* = 0.038), IgG1 (> 6.22 g/liter, *P* = 0.025), IgG2 (> 2.42 g/liter, *P* = 0.019), and IgG3 (> 0.21 g/liter, *P* = 0.034) were significantly and positively correlated with PFS, whereas OS prolongation was only notably relevant with elevated IgG2 subclasses (34).

Additionally, measurement of pre-treatment circulating autoantibodies in melanoma patients treated with ICI revealed that elevated anti-MAGEB4 levels were linked to longer OS and development of irAEs, while high levels of pre-treatment anti-FGFR1 antibodies were linked to shorter OS and lower frequency of irAEs (35).

### 3.2 Inflammation storm

Not only the changes in the immune cell profile, but also the inflammatory storm caused by systemically activated pro-inflammatory cytokines. Eleven circulating cytokines, known as G-CSF, GM-CSF, Fractalkine, FGF-2, IFN-α2, IL-12p70, IL-1a, IL-1B, IL-1RA, IL-2, and IL-13, were dramatically upregulated in melanoma patients with severe irAE at baseline and early during combined ICI treatment, and were integrated into a new cytokine toxicity score (36).

#### 3.2.1 IL-1

A higher expression profile of the NLRP3 inflammasome was observed in bone marrow cells of patients with immune-associated colitis, whose activation triggers IL-1 release, suggesting that IL-1 may be involved in the development of colonic inflammation (25).

With combined blockade of CTLA-4 and PD-1, colitis-related symptoms such as diarrhea and weight loss were not identified by Andrews et al. (16) in the mouse model, but subclinical toxicity like shortened ileal villi, mucosal damage and inflammatory infiltration were uncovered. Transcriptional analysis of the ileum verified rapid Transcriptional upregulation of IL-1b, rather than TNF-α or IL-6. After tentatively addition of IL-1R antagonist, the intestinal inflammation was alleviated noticeably, hinting the gut microbiome mediates ICI-induced intestinal toxicity via IL-1β. The specific mechanisms underlying this are, as yet, unclear.

#### 3.2.2 IL-6

IL-6 is a key factor in the differentiation of CD4^+^ T cells into Th17. IL-6-induced inflammation was observed in patients who developed immune-related enterocolitis (irEC) after treatment with ICI, as evidenced by more incredible upregulation than normal tissue of acute phase reactants (e.g. IL-11), genes encoding neutrophil and monocyte chemotactic molecules (e.g. CXCL1, CXCL2, CXCL3 and CXCL8) and neutrophil count (37). Surprisingly, there was no apparent elevation of Th17 memory cells in patients treated with anti–PD-1 therapy, while completely different from anti–CTLA-4 therapy with abundant Th17. Therefore, inflammation mediated by IL-6–Th17 pathway can probably drive the progress of irEC, so that addition of IL-6 blockers to tumor-bearing mice treated with anti–CTLA-4 reduces Th17, macrophages counts and tumor load, but promotes tumor shrinkage and increases survival rate of mice (37).

#### 3.2.3 IFN signaling pathway

T cell – myeloid crosstalk relies on the IFN signaling pathway.

**Figure 1:**
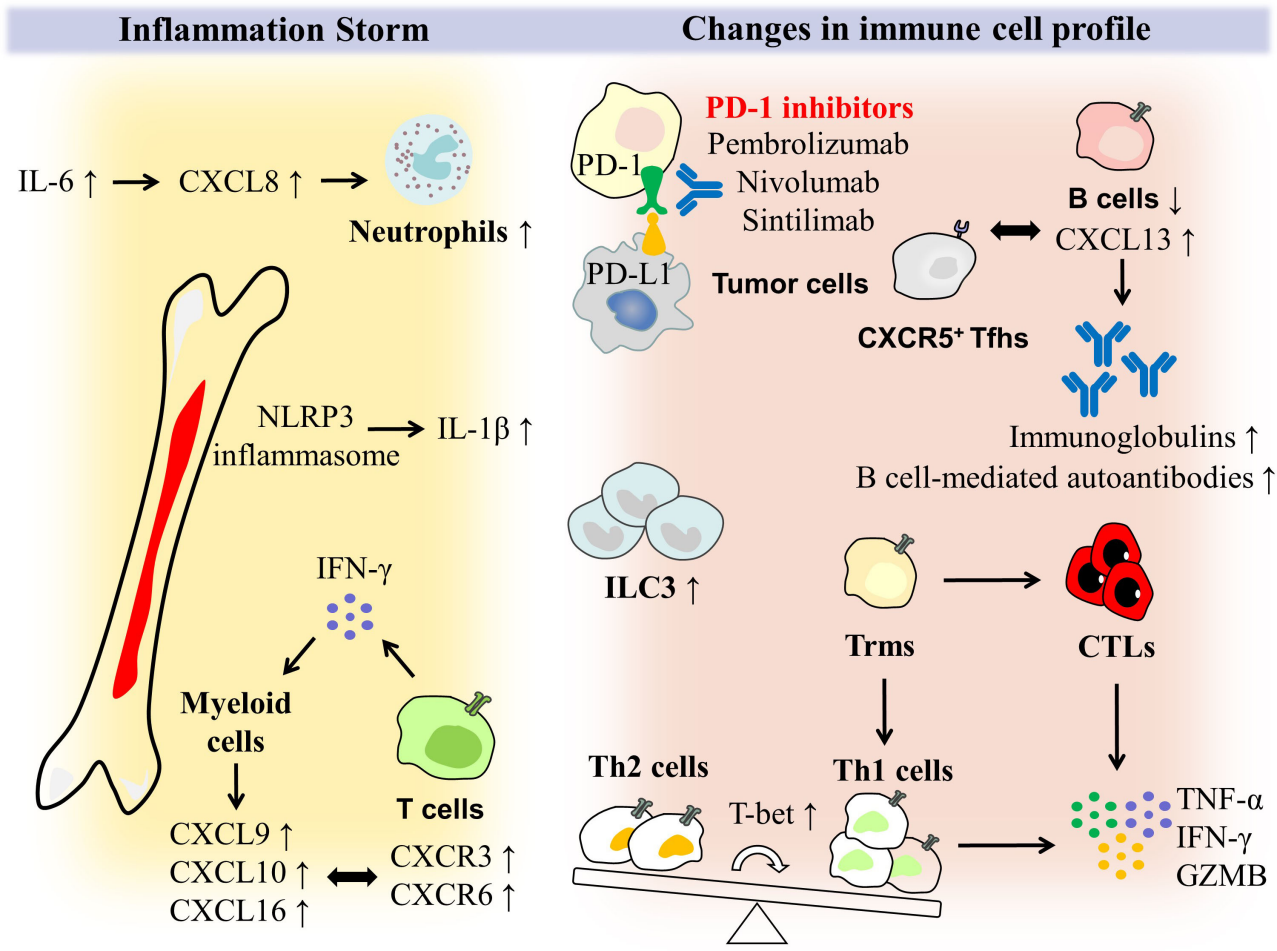
Potential mechanisms of immune cells and inflammatory storm in PD-1 induced colitis. PD-1 expressed by T cells can bind to PD-L1 ligands activated by tumor cells, thereby inhibiting T cell activation. PD-1 inhibitors such as pembrolizumab, nivolumab and sintilimab block these processes to enhance the T cell anti-tumor response. With high expression of T-bet, the balance of CD4^+^ T cells is tilted toward Th1 cells, which is dominant compared with Th2 cells. Moreover, tissue-resident memory T cells (Trms) rapidly differentiate into CTLs and Th1 cells, and secrete the pro-inflammatory factors like TNF-α, IFN-γ and GZMB. ILC3 was enriched in intestinal mucosal tissues. As irAEs occur and grow in severity, the absolute number of B cells decreases. However, CXCR5+ T follicular helper cells (Tfhs) accumulate in the germinal centers and plasma expression of the B lymphocyte chemokine ligand CXCL13 increases, ultimately allowing for B cell-mediated overproduction of autoantibodies and immunoglobulins. In PD-1-induced colitis, there is not only a change in the immune cell profile, but also an onslaught of inflammatory storms. NLRP3 inflammasome expression and induction of IL-1β release are found to be higher in myeloid cells. IL-6 induces an increase in CXCL8, which encodes a neutrophil chemotactic molecule, giving rise to the number of neutrophils. IFN-γ plays an essential role in the interaction between T cells and myeloid cells, as it stimulates high expression of CXCL9, CXCL10, and CXCL16 in myeloid cells, allowing T cell populations expressing CXCR3 and CXCR6 chemokine receptors to be rapidly recruited to inflamed tissues and secrete IFN-γ, which eventually formed a closed loop of the IFN signaling pathway.

IFN-γ induces hyper-expression of CXCL9, CXCL10 (ligand for CXCR3) and CXCL16 (ligand for CXCR6) in bone marrow cells of patients with colitis, which then encodes chemokines that recruit effector T cells to the site of inflammation; accordingly, expression of chemokine receptor genes CXCR3 and CXCR6 is upregulated in the T cell population (25). The molecular mechanism of T cell recruitment may be a therapeutic target, for example, blockade of CXCR3 or CXCR6 could theoretically ameliorate cancer metastasis and reduce intestinal inflammation (**Figure 1**).

### 3.3 The role of gut microbes

In patients treated with anti–CTLA-4, their baseline gut microbiota was found to be associated with an elevated susceptibility to ICI antitumor response and to the development of enterocolitis (6). Although anti–PD-1 drugs have milder gastrointestinal irAEs compared with anti–CTLA-4 (9), a growing attention has been paid to the gut microbiome of patients treated with PD-1 inhibitors. How the microbiota and their metabolites affect anti–PD-1 efficacy and gastrointestinal adverse outcomes is gradually being unveiled.

#### 3.3.1 Changes in the gut microbial spectrum

Scholars have identified a lot of overlap with microorganisms that are highly responsive to immunotherapy and those involved in prevention of irAE development. Some bacterial species such as *Akkermansia muciniphila* (*A. muciniphila*), *Bacteroides fragilis*、*Bifidobacterium* spp., *Faecalibacterium* spp. (especially *F. prausnitzii*) and *Ruminococcaceae* spp.are involved in favorable antitumor immune responses (38, 39). Patients who responded poorly to ICI treatment and suffered from grade 3 or higher severe adverse reactions had significantly lower microbiome diversity at baseline (39). Therein, declined relative abundance of *A. muciniphila*, *F. prausnitzii* and *Ruminococcaceae* family was observed in the intestine of patients with severe irAE (39). *Bacteroides intestinalis* and *Intestinibacter bartlettii* were observed to be enriched in melanoma patients who developed grade 3 and higher irAEs; while *Anaerotignum lactatifermentans* and *Dorea formicigenerans* were enriched in patients with low-grade toxicity (16). Likewise, Zhang et al. (40) analyzed the gut microbiota profiles of 23 patients with gastrointestinal cancer who received immunotherapy recently, finding that species such as *Clostridium hathewayi*, *Ruminococcus torques* and *Megamonas* were enriched in patients without irAEs; inversely, *Bifidobacterium dentium*, *Rothia mucilaginosa* and *Gemella haemolysans* were significantly higher in irAE patients. Moreover, *Ruminococcus callidus* and *Bacteroides xylanisolvens* were enriched in patients without severe irAEs, which helps to distinguish the population with ≥3 irAE.

Assessment of fecal microbiota composition by 16S rRNA gene sequencing revealed that the abundance of the phylum *Bacteroidetes* was negatively associated with anti-CTLA4–associated colitis, in which patients enriched are less likely to develop colitis perhaps due to expression of polysaccharide A can induce Tregs to prevent colitis; while patients enriched in *Faecalibacterium* genus and phylum *Firmicutes* are more likely to develop colitis, although with longer PFS and OS (23, 41). In the same way, stool samples from lung cancer patients were tested and *Raoultella* were discovered to be overrepresented in the feces of patients who undergo less serious irAE; whereas *Agathobacter* was linked to more severe irAE profile, although it involved in beneficial clinical outcomes (42).

Of concern, the classic probiotics *Lactobacillus* and *Bifidobacterium* play an important role. Experiments by Wang and companions demonstrated a significant decrease in the relative abundance of *Lactobacillus* in severe ICI-associated colitis. After complete clearance of *Lactobacillus* with antibiotic vancomycin, the mice immediately displayed obvious weight loss, severe inflammatory cell infiltration and elevated levels of pro-inflammatory cytokines such as TNF-α, IL-6 and IFN-γ. Instead, all these manifestations were noticeably improved via reducing in ILC3s after oral administration of probiotic *Lactobacillus reuteri* adequately supplemented with *Lactobacillus*, defined as no further weight loss, less inflammatory cell infiltration, and partial restoration of colonic structure in mice. It is noteworthy that there was no visible difference in the count of tumor-infiltrating T cells and the growth rate of tumors as well as the OS of mice was not affected. In summary, they highlight the intestinal microorganism *Lactobacillus* can symptomatically attenuate ICI-induced immune-mediated colitis without affecting the efficacy (28). It is quite remarkable that Sun et al. (43) revealed *Bifidobacterium*, one of the well-known probiotics, was able to induce microbiome optimization, such as an increase in the proportion of *Lactobacillus*, after colonizing the gut of mice. This altered commensal community enhanced the IL-10–mediated inhibitory function of intestinal Tregs, which contributed to the mitigation of colitis in mice in the context of CTLA-4 blockade. When dual immune checkpoint blockade was performed, Tan et al. discovered *Lactobacillus rhamnosus GG* alleviated severity of irAEs colitis in mouse models, as manifested by significantly reduced disease activity index, histopathological score and CD8+ T cell counts, together with increased FoxP3^+^ Tregs (44).

Perhaps other technologies such as macrogenome sequencing can be applied in the future to better characterize the entire gut microbiome and clarify its relationship between clinical benefit and adverse effects of ICI treatment.

#### 3.3.2 Metabolites of intestinal flora

Accumulating studies have led scholars to speculate that the poor outcome of immunotherapy is connected to the decrease in beneficial microbiome metabolites, including B-vitamins synthesis, inosine as well as short chain fatty acid (SCFA) production (39).

##### 3.3.2.1 B-vitamins

The gut microbiota produces bioactive compounds including B-vitamins, which have been reported to act not only as nutrients but also as modulators of colitis (45). For instance, vitamin B3 (niacin) deficiency leads to intestinal inflammation and diarrhea, while proper supplementation with vitamin B6 can alleviate IBD (46).

##### 3.3.2.2 Bacterial-derived metabolite inosine

Following the action of ICIs, the metabolite inosine produced by *A. muciniphila* and *B. pseudolongum* promotes the activation of anti-tumor Th1 cells through T-cell–specific A_2A_ receptor signaling in response to ICIs immunotherapy (47). Hereafter, the efficacy of the metabolism and recovery of inosine degradation products including xanthine and hypoxanthine in immunotherapy deserves further study.

##### 3.3.2.3 Short chain fatty acid

SCFA includes propionate and butyrate. Propionate and butyrate can improve intestinal barrier function, accelerate the repair of intestinal epithelial cell damage and maintain intestinal homeostasis (48, 49). In the analysis of gut microorganisms from patients’ fecal samples with gastrointestinal cancers treated with anti–PD-1/PD-L1 therapy, there were overrepresented commensal SCFAs-producing bacteria, including *Eubacterium*, *Lactobacillus*, and *Streptococcus* in patients with satisfactory outcomes (50). Zhang et al. observed an enrichment of *Eubacterium rectale* and *Megasphaera elsdenii*, in non-/low irAEs gastric cancer patients (40).

SCFA is the main fermentation product of dietary fiber. Turning our attention to eating habits, dietary fiber intake affects the immune function of the gut microbiota and the development of irAEs. Propionate levels are significantly elevated in melanoma mice on a fiber-rich diet (51). In the case of high abundance of butyrate-producing *Ruminococcaceae*, researchers found that the corresponding hosts positively responded to dietary nutrient intake, i.e., displayed high fiber and omega 3 fatty acid consumptions (39). Consequently, a high dietary can be responsible for increase in *Ruminococcaceae* to support the maintenance of intestinal integrity; on the contrary, a low dietary is easily susceptible to poor response and adverse outcomes in immunotherapy.

**Figure 2:**
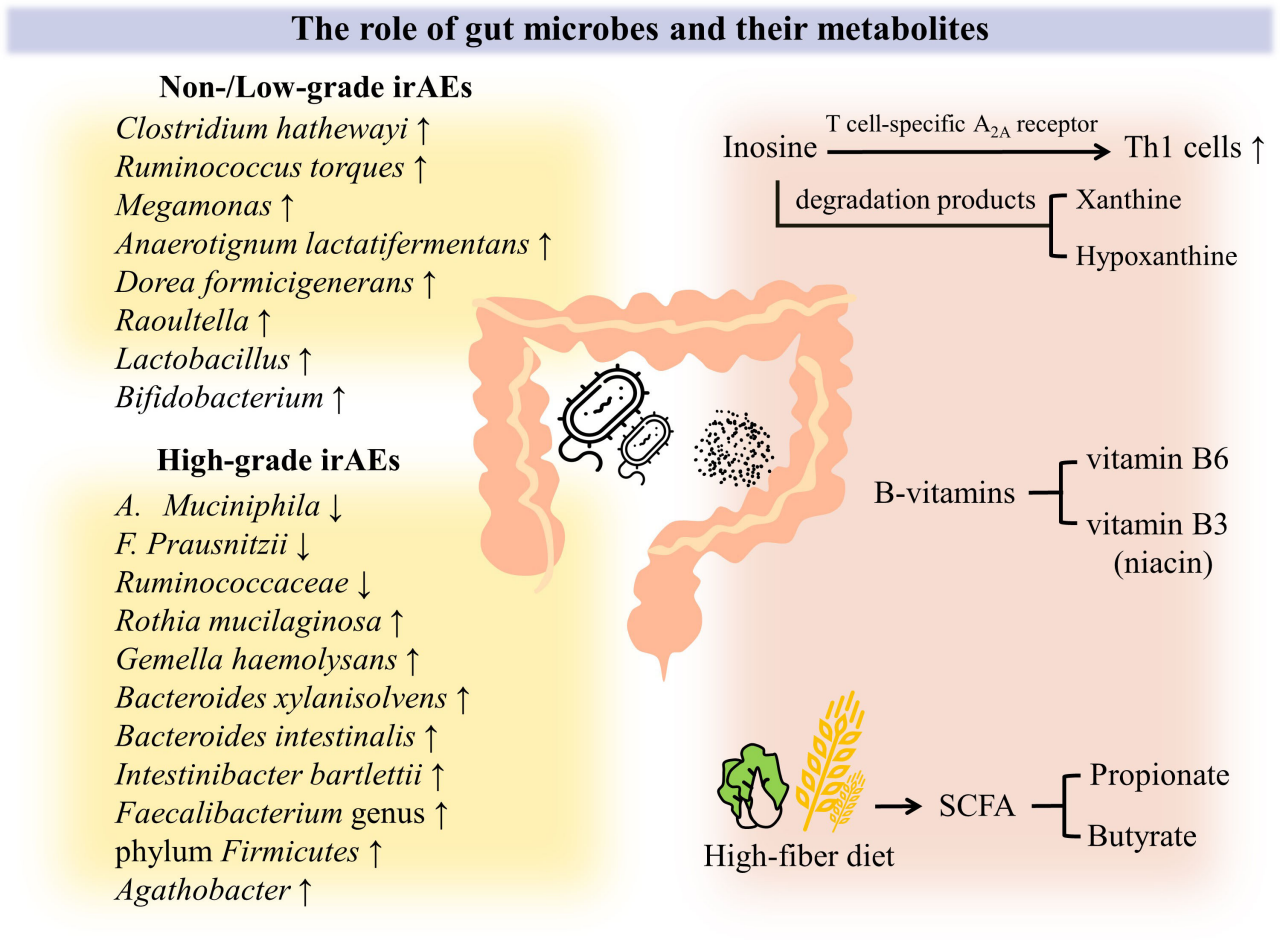
Crosstalk between gut microbiome and PD-1 induced colitis. A growing body of evidence demonstrates that intestinal flora and its metabolites play an influential role in the occurrence and varying severity of irAEs. First of all, the intestinal microbial spectrum has changed. Even with the heterogeneity of tumor types and dosing regimens, Clostridium hathewayi, Ruminococcus torques, Megamonas, Anaerotignum lactatifermentans, Dorea formicigenerans, Raoultella, Lactobacillus and Bifidobacterium was found to be abundant in non-/low-grade irAEs patients. When it comes to high-grade irAEs, declined abundance of A. muciniphila, F. prausnitzii, Ruminococcaceae was observed, but Rothia mucilaginosa, Gemella haemolysans, Bacteroides xylanisolvens, Bacteroides intestinalis, Intestinibacter bartlettii, Faecalibacterium genus and phylum Firmicutes, Agathobacter was found to be enriched. In addition, the role of intestinal flora metabolites has been increasingly appreciated. B vitamins such as vitamin B3 and B6 can alleviate intestinal inflammation to some extent. Inosine enhances the antitumor effect by promoting Th1 cells activation through T-cell–specific A2A receptor, and even its degradation products xanthine and hypoxanthine may play a role. Gut microbial-dependent dietary interventions are currently emphasized, as high dietary fiber promotes an increase in the proportion of SCFA-producing bacteria, resulting in significantly higher levels of SCFA (e.g., propionate and butyrate), which can effectively reduce intestinal inflammation and maintain intestinal homeostasis.

When Spencer et al. evaluated melanoma patients treated with anti–PD-1, they revealed that patients with adequate dietary fiber intake had a high microbial alpha diversity with abundant *Ruminococcaceae* family and *Faecalibacterium* genus as well as a prolonged PFS, even if not statistically significant. Subsequently, they performed corresponding parallel animal experiments. Melanoma mice with anti–PD-1 therapy exhibited tumors delayed growth after a high-fiber diet, accompanied by increased CD4^+^ T cells; in contrast, mice receiving a low-fiber diet had significantly fewer IFN-γ ^+^ CD8^+^ T cells for mice with impaired treatment response to anti-PD-1–based therapy. It should be emphasized that none of these results could be observed in germ-free mice. It is suggested that dietary intervention for tumor immunotherapy is gut microbial-dependent and that fiber contribute to the immunotherapy by gut flora affecting T cell activation. Therefore, we urge oncology patients with PD-1 inhibitors therapy to pay more attention to dietary habits in daily life (51) (**Figure 2**).

## 4 Treatment strategies

### 4.1 Pre-treatment preparation

Intestinal inflammatory markers such as fecal lactoferrin and calprotectin should be performed as part of the initial workup, which is critical to determine disease activity and guide therapeutic intervention. Meanwhile, patients with G2 and higher grades diarrhea/colitis symptoms should undergo fecal infectivity analysis to rule out infections such as *Clostridium difficile* and cytomegalovirus (CMV) in addition to whole blood PCR for routine blood count and serum CRP. Clinical suspicion of celiac disease was clarified by serum anti-tissue transglutaminase immunoglobulin (tTG-IgA).

Approximately 95% of patients have ICI-induced inflammation in the left colon on biopsy (52). Recently, a cross-sectional study organized by De Silva et al. uncovered 93.8% of left-sided colon (defined as distal 25 cm of the colon, rectosigmoid colon) biopsies with an abnormal endoscopic appearance and, to the surprise, up to 68.6% of normal-appearing mucosal biopsies with histological evidence of colitis (53). They finalize by noting that extensive biopsies of left-sided normal and abnormal segmental mucosa for evaluation of immune-mediated colitis with flexible sigmoidoscopy hold promise as a simplified alternative to full colonoscopy, although poor sensitive but extremely specific and is often sufficient for early initial diagnosis or follow-up review to guide treatment strategy. Thus, flexible sigmoidoscopy or colonoscopy with biopsy is strongly recommended within 2 weeks of onset for all G2patients and G1patients with positive lactoferrin.

### 4.2 Symptomatic treatment

For patients with G1 diarrhea/colitis, in case of a negative lactoferrin result and no infection, symptomatic supportive management including a low-fiber dietary combined with the antidiarrheal drugs such as loperamide, if necessary, mesalamine or cholestyramine may be administered.

### 4.3 Corticosteroid

The cornerstone of toxicity management is often immunosuppressive agents, which is widely used as a first-line treatment. However, the side effects of their long-term use have been demonstrated such as adrenal insufficiency and osteoporosis, particularly the increased risk of opportunistic infections (54), and even high dose of glucocorticoid (≥ 60 mg prednisone equivalent once a day) in early onset severe irAEs (within 8 weeks of anti–PD-1 initiation) has recently been implicated in compromising the antitumor efficacy of PD-1 inhibitors, resulting in poorer PFS and OS in patients (55). In view of its double-edged sword effect, the use of hormones should be individualized after a comprehensive assessment of patient’s responsiveness and tolerance. Pay attention to the past medical history of patients, preferably the elderly with diabetes or in an immunocompromised state (54). Use the lowest effective dose of corticosteroids within the shortest possible time.

To optimize follow-up management strategies, it is imperative to focus on endoscopic and histologic features for their early suggestive role. Wang et al. elucidated that, in patients with endoscopically ulcers, there was a clinically higher frequency of steroid-refractory colitis (*p* = 0.044) and severe diarrhea (*p* = 0.033) (11). In light of this finding, clinicians may administer stronger immunosuppressive agents early in patients with endoscopic ulcers to avoid poor steroid response.

### 4.4 TNF-blocking antibodies

Since the expression of TNF in the gut is evidently elevated in patients with PD-1 inhibitor-induced colitis, it is theoretically feasible to target checkpoint-induced colitis with TNF blockade. Some scholars even laughingly describe anti-TNF as a magic bullet.

A retrospective study investigated the outcome of infliximab (IFX) therapy in eight patients of melanoma and lung and kidney cancers with severe steroid-resistant irAEs induced by ICIs. IFX was found to be effective in relieving colitis in these patients by neutralizing TNF-α, leading to the conclusion that early infusions of IFX in combination with systemic steroid therapy is necessary (56).

Etarnercept, known as a TNF-α cytokine-targeted antagonist, is widely used in the treatment of rheumatoid arthritis with outstanding efficacy and safety (57). When adding etarnercept to the colon cancer mice model with co-treatment of nivolumab and ipilimumab, Perez-Ruiz et al. noticed tumors remained well-controlled with no reduction in efficacy but ICIs-aggravated colitis was significantly alleviated (58).

The above experiments suggest the combined use of anti-TNF with ICIs may have a positive effect on efficacy and safety. It is reasonable to prophylactically neutralize TNF and appropriately increase the blood concentration of TNF antagonists to ensure low adverse reactions. We look forward to more real-world research conclusions to guide drug usage.

### 4.5 Vedolizumab

As a gut-selective α4β7 integrin antibody, vedolizumab (VDZ) inhibits T-cell migration to inflamed gastrointestinal tissues by blocking its binding to the gut mucosal addressin cell adhesion molecule 1 (MAdCAM-1). VDZ is approved for widely long-term use in inflammatory bowel diseases (59). After exploration by scholars, VDZ is also capable of effectively inhibiting ICIs-related corticosteroid-refractory gastrointestinal toxicity without hindering the antitumor effects (5).

In terms of the comparative efficacy of IFX and VDZ in immune-associated colitis, a multicenter study surrounding the role of VDZ therapy in ICI-induced steroid-refractory colitis was conducted under the direction of Abu-Sbeih et al. After infusions of VDZ to patients who had been treated with steroids and/or IFX with poor results, approximately 86% of patients achieved clinical remission, 54% achieved endoscopic remission, and 29% achieved histologic remission (60). A two-center, retrospective study involving 184 patients (62 VDZ, 94 IFX, and 28 combination) clarified that there was similar oncological outcomes and clinical remission of colitis, but VDZ had a shorter duration of steroid therapy, fewer hospitalizations, lower recurrence rates of colitis than IFX, with the exception of a longer time to clinical response (61). As a consequence, VDZ is a potent alternative because of intestine-specific mechanism and efficacy for primary non-responders to anti–TNF-α.

Abu-Sbeih et al. pointed out that, analogous to the management protocol for IBD, selective immunosuppressive therapy (SIT) with IFX or VDZ monoclonal antibodies should be introduced early in the course of immune-mediated colitis (i.e., within 10 days of the onset), regardless of the response to corticosteroid therapy, as it allows for a shorter duration of clinical symptoms and fewer hospitalizations, while helping corticosteroid steroid dose reduction smoothly. SIT three and more infusions resulted in a better frequency of histological remission, lower fecal calprotectin levels, and more importantly, a distinct reduction in the recurrence rate of colitis (62). However, the heterogeneity of tumor types and dosing regimens of participants enrolled in the cohort makes this conclusion not equally applicable to all types of ICI-induced colitis, pending validation in prospective studies with larger samples.

It must be mentioned that, in spite of the positive feedback from patients on the combination of corticosteroids and biologics in many current studies, there is insufficient evidence on the risk and influence of OS in the immunocompromised advanced cancer population.

Several guidelines currently go into detail on these three weapons (corticosteroid, IFX, and VDZ), including the American Society for Clinical Oncology (ASCO), the European Society for Medical Oncology (ESMO), the National Comprehensive Cancer Network (NCCN), and a prior consensus statement from the Society for Immunotherapy of Cancer (SITC) (4, 63–65). Oral 1 mg/kg per day prednisone is recommended for G2 patients and G1 patients with positive lactoferrin as well as persistent or progressive diarrhea symptoms until symptoms improve significantly, followed by a taper over 4–6 weeks. Introduction of biologics within 2 weeks in addition to corticosteroids may be considered for those whose colitis is corticosteroids-refractory (i.e., no decrease by one grade in 3 days) or with high-risk endoscopic ulceration. IFX stands out as a preferred agent for those who do not respond to acute severe colitis, whereas VDZ is used as an alternative due to a mild delayed response. Three doses of IFX (5 mg/kg) are given at weeks 0, 2, and 6. If symptoms persist after the second dose of IFX administration, the third dose should be suspended and accordingly 3 doses of VDZ (300 mg) should be provided at weeks 0, 2, and 6. Repeat endoscopy to assess mucosal healing and fecal calprotectin surveillance may guide the timing of biologic dosing. Of note, to avoid reactivation of latent tuberculosis infection, VDZ is advisable rather than IFX for patients with tuberculosis, in which case the patient ought to obtain TB test before the first dose of biologics. Inpatient monitoring is strongly recommended for G3 and G4 patients. An intravenous prednisone or methylprednisolone should be administered at an initial dose of 1–2 mg/kg per day, followed by tapering over 4–6 weeks until symptom improve to G1 and switching from intravenous to oral at the appropriate time. If there is inadequate response after 3 days, IFX or VDZ may be added the same as G2. For patients with symptoms including fever, abdominal pain, and blood in the stool, abdominal CT scan should be performed immediately in order to rule out suspected complications and treat as soon as possible.

### 4.6 IL-1R antagonist

Current research indicates that targeted therapy with IL-1 receptor antagonists in autoimmune diseases such as Still’s disease improves clinical outcomes (66). As mentioned above, the cytokine IL-1 performs an important part in the development of immune-related gastrointestinal adverse reactions. On the basis of this theory, Andrews et al. found with anakinra was sufficient to reverse dual ICIs-induced ileitis by blocking IL-1β without compromising the therapeutic efficacy in mice (16).

### 4.7 IL-6R inhibitor

Extensive attention has been drawn to clinical data that anti–IL-6 receptor monoclonal antibody (i.e., Tocilizumab and Sarilumab) is effective in high-level steroid-refractory colitis secondary to PD-1 blockers, while not compromising its efficacy. Thus, IL-6 antagonist may be a promising option for the steroid refractory irAEs with manageable safety profile.

After treatment with nivolumab, patients who developed severe colitis had a multiplicative increase in CRP from baseline. Given poor results with corticosteroids alone, Stroud et al. (14) added tocilizumab and noticed clinical remission followed by CRP decline to baseline or even lower. However, owing to the small sample size of this research and the lack of randomized trials to demonstrate the efficacy of Tocilizumab and its effect on PD-1 blocker therapy, further studies are desperately needed.

Similarly, Hailemichael et al. (37) retrospectively analyzed nearly 14,000 patients with melanoma, most of whom developed refractory irAEs after treatment with PD-1 blockers. With the addition of tocilizumab or Sarilumab to those who failed to improve after corticosteroids, the inflammatory indicators such as CRP and inflammatory cells were reduced; moreover, the significant increase in the assessment of patients’ overall response rate to ICI revealed that IL-6 antagonists could enhance tumor immunity and attenuate the corresponding toxicity. It is a pity that there were no patients with gastrointestinal side-effects secondary to PD-1 therapy, so more experimental results are strongly expected.

Later Holmstroem et al. (67) revealed that Tocilizumab had a clinical benefit rate of up to 84% in ICI-induced colitis. By recruiting patients with CTCAE v5.0 grade > 1 ICI-induced colitis/diarrhea and adding Tocilizumab to non-stop ICI while refusing hormones or immunosuppressants, they observed that the vast majority of patients achieved clinical remission (CTCAE reduction ≥ grade 1) within 8 weeks and hormone-free remission within 24 weeks.

However, contradictory to the above conclusion, preventive blockade of IL-6 in mice with tumors derived by subcutaneous transplantation of human MC38 colon cancer cells or B16-ovalbumin, anti-tumor efficacy of anti–PD-1 and anti–CTLA-4 combination in mice was partially reduced in case of Perez-Ruiz et al. (58).

Thus, we required further studies to confirm the role of IL-6 blockades in gastrointestinal toxicity and the optimal control dose.

### 4.8 Janus kinase inhibitor tofacitinib

The efficacy of Janus kinase (JAK) inhibitors stands out in the various inflammatory diseases, especially the potent JAK-selective inhibitor tofacitinib, currently found to serve as induction and maintenance therapy for ulcerative colitis (68). Due to the similarity of disease manifestations, its role in anti-PD-1–caused colitis has been explored.

A patient with gastric cancer developed colitis after pembrolizumab, presenting as refractory diarrhea, which was insensitive to successive glucocorticoids, infliximab and vedolizumab, and even if there was improvement, it recurred after dose reduction or even remained ineffective after increase in dose, while Esfahani and his colleague noticed that the diarrhea improved rapidly with the addition of third-line treatment with tofacitinib (69). Maybe tofacitinib act as a promising target with favorable outcomes and a good safety profile in refractory immune-related colitis.

### 4.9 Fecal microbiota transplantation

Gut microbes and their metabolites perform an unexpected function in cancer immunotherapy. We are looking forward to further breakthroughs with PD-1 inhibitors by reshaping microbes to enhance efficacy but minimize toxicity. The approach of fecal microbiota transplantation was then proposed and is proven by a growing number of scholars for its feasibility in dealing with PD-1 inhibitor-induced organ toxicity.

A patient with metastatic uroepithelial carcinoma developed grade 2 diarrhea/colitis after combination blockade of CTLA-4 and PD-1 and colonoscopy indicated severe colitis resembling ulcerative colitis. Another patient with prostate cancer received eprimar was hospitalized for fever and colitis and colonoscopy suggested Crohn’s-like manifestations of ICI-associated colitis. After no relief with systemic corticosteroids, infliximab, and vedolizumab, they were treated with intestinal flora transplantation from a healthy donor, noticing rapid resolution of clinical symptoms and significant improvement in endoscopic evaluation, including regression of ulcers and reduction in inflammatory immune cell infiltration. Simultaneously, the type of bacteria with a protective effect was enriched after FMT treatment. The first patient was predominantly *Clostridia*, with a marked lack of *Bacteroidia* and *Verrucomicrobiae*. After FMT, there was a greater number of *Clostridia* and a significantly higher accumulation of *Akkermansia*, as well as an amplification of *Bifidobacterium*. The second patient was predominated by *Gammaproteobacteria*, such as Escherichia. After conducting FMT, the abundance of *Blautia* and *Bifidobacterium* increased noticeably. Puzzlingly, it was a gradual improvement of gastrointestinal symptoms, but the number of potentially pathogenic *Escherichia* first decreased and then increased significantly, while the beneficial *Bacteroides* increased and then eventually appeared to decrease. This case report delivered by Wang et al. highlights the role of FMT in rapidly and safely eliminating ICI-associated colitis after reconstitution of the gut microbiome (70).

Later, in a phase I clinical trial, fecal microorganisms from two patients, who had been previously treated with PD-1 inhibitors and achieved complete remission (CR) for more than 1 year, were transplanted to 10 patients who had failed anti–PD-1 therapy and had been depleted of intestinal flora by oral antibiotics. Then anti–PD-1 therapy was re-induced, and three of 10 persons showed clinical responses, including 1 CR. Analysis of their post-treatment tumor samples revealed upregulation of multiple immune-related genomes, such as IFN-γ–mediated signaling pathways, T-cell activation, and Th1 immune response. Although this study could not specify the flora profile that induces a clinical benefit for the time being, FMT and anti–PD-1 reprogrammed the TME and safely overcame immunotherapy resistance by modulating the intestinal flora (71).

FMT may become a first-line treatment when more evidence is evaluated. However, by means of FMT to overcome the anti-PD-1–caused toxic effects is not available to all institutions. Furthermore, there is insufficient evidence to guide the optimal parameters of FMT at present, such as donor requirements, operating technique, frequency, and applicable cancer types. Even though it is mechanistically possible to be an efficient therapeutic target, a variety of difficulties will make this novel therapy option challenging to spread.

### 4.10 The application of traditional Chinese medicine

The concept of “nourishing positive accumulation and eliminating cancer by itself” in traditional Chinese medicine (TCM) probably has the concept of eliminating tumors by restoring the body’s immune system, which is identical to the tumor killing mechanism of PD-1 inhibitors (72). Increasing emphasis is being placed on TCM that act as immune checkpoint modulators (73). For instance, in combination with PD-1 inhibitors, ginsenoside Rg3 can function in diffuse large B-cell lymphoma and juzentaihoto (Shiquan Dabu Decoction) can function in B16 melanoma cancer (72). Mounting studies have demonstrated that TCM can modulate the TME and related effectors to influence clinical outcomes.

Baicalin, a bioactive component of Scutellaria baicalensis, is capable of reducing the levels of inflammatory factors IL-1β, TNF-α, and IL-6, as well as blocking the PD-1 signaling pathway by inhibiting IFN-γ, thereby playing an essential role in anti-inflammation and anti-tumor efficacy of PD-1 antibodies (74).. Of note, baicalin promotes an increase of intestinal bacteria producing SCFA (especially butyrate), which may theoretically inhibit the occurrence of irAEs (74).

Meng et al. (75) found that PD-1 inhibitors in melanoma mice may lead to an increase in Th2 cells and secretion of IL-4, IL-5, and IL-10, which have a negative effect on antitumor effects. The addition of Brucea javanica oil emulsion (BJOE) to anti–PD-1 revealed not only successfully reversed the suppressed TME with reduced Tregs levels but also showed significant synergistic anti-tumor effects with sound biosafety, as manifested by a notable inhibition of tumor growth, improved survival rate in mice without weight loss, and an increase in CD4^+^ T cells, CD8^+^ T cells and the proportion of MI/M2 macrophages as well as an increase in TNF-α, IFN-γ, CXCL10, and granzyme B.

When Gegen Qinlian decoction, constituted from *Radix Puerariae*, *Scutellariae Radix*, *Coptidis Rhizoma*, and *liquorice* in certain proportions, was combined with anti-mouse PD-1 in mouse models of colorectal cancer, Lv et al. noticed that tumor growth was significantly inhibited, intestinal flora *Bacteroidales_S24-7_group* was remarkably abundant, as well as increased levels of CD8^+^ T cells and IFN-γ in tumor tissues and decreased levels of PD-1 (76). This combination therapy enhances anti-colorectal cancer activity when it fails to respond to anti–PD-1 agents, by reshaping the intestinal flora and restoring T-cell function.

Overall, since the mechanism of herbs enhance anti-tumor activity without adverse effects as mentioned above, perhaps its combination therapy with anti–PD-1 can be popularized in more cancer patients.

### 4.11 Artificial intelligence

AI is penetrating into our daily life, including a growing role in cancer immunotherapy. Scholars found to their surprise that AI can be prospectively used for immunotherapy response prediction. Predictive models are built by AI combining with big data, including radiomics, genomics, proteomics, transcriptomics, pathology imaging, and clinical characteristics (77). This AI-based clinical decision-making system promotes circumventing differences caused by heterogeneity of tumor immune microenvironment and identifying patients who benefit most from ICI with minimal adverse clinical effects, so as to help clinicians develop individualized treatment plans (78).

Although AI has yet to mature further and require large public database of different centers to improve accuracy, we can be confident that, in the future, it can help build powerful predictive models and shine in the field of precision oncology (**Table 2**).

**Table 2:** Multiple treatment strategies for addressing irAEs.

Therapeutic methods	Specific details
Pre-treatment preparation	Detection of intestinal inflammatory markers like fecal lactoferrin and calprotectin; Screening for Clostridium difficile and CMV infections in feces; Determination of celiac disease by tTG-IgA; Early use of flexible sigmoidoscopy or colonoscopy with biopsy (53).
Symptomatic treatment	A low-fiber dietary; Antidiarrheal drugs like loperamide; Add mesalamine or cholestyramine if necessary.
Corticosteroid	Individualized hormone application; Use the lowest effective dose of corticosteroids for the shortest period of time (54).
TNF-blocking antibodies	Combined use of ICIs with anti-TNF agents such as infliximab or etarnercept (56, 58).
Vedolizumab	Vedolizumab therapy in ICI-induced steroid-refractory colitis or non-responders to anti–TNF-α (60, 61); Early introduction (within 10 days of the onset) of VDZ and sustained 3 or more infusions (62). Available for tuberculosis patients.
IL-1R antagonist	Reversal of dual ICIs-induced ileitis with anakinra (16).
IL-6R inhibitor	Use of Tocilizumab or Sarilumab for refractory irAEs (14, 37, 67).
Janus kinase (JAK) inhibitor	Application of the potent JAK-selective inhibitor tofacitinib in refractory immune-associated colitis (69).
Fecal microbiota transplantation (FMT)	Remodeling of the gut microbiota by receiving intestinal flora from a healthy donor (70, 71).
Traditional Chinese Medicine	Joint application anti–PD-1 drugs with Baicalin (74), Brucea javanica oil emulsion (BJOE) (75) and Gegen Qinlian decoction (76).
Artificial intelligence (AI)	As an assistive technology, AI can be combined with big data to build an AI-based clinical decision-making system for minimal adverse reactions (77, 78).

## 5 Predictive biomarkers

Scholars are devoted to investigate biomarkers that can predict gastrointestinal toxicity early, which are mentioned in **Table 3**. Nevertheless, tumor heterogeneity may result in restrictions on the application of biomarkers. A greater number of prospective studies are required to put them into clinical practice.

**Table 3:** Exploration of biomarkers for early prediction of irAEs.

Biomarkers	High risk of irAE occurrence	Low risk of irAE occurrence
Blood counts and biochemical indicators	Lower NLR (< 3) and PLR (< 180) as well as higher AEC (> 240/μl) at baseline (79); Performance status (PS) ≥ 2 and albumin levels < 35 g/liter (80).	
Genetic susceptibility	The presence of HLA-DQB1*03:01 (81); Higher expression levels of LCP1 and ADPGK (17); Mutations in the NRAS gene (*p* = 0.2844) (82).	Mutations in the oncogenic gene BRAF (*p* = 0.0180) (82); With the SNPs (CYP24A1 rs2762934, CYP27B1 rs10877012, and SEC23A rs8018720 genotypes) (83).
Dynamic circulating tumor DNA (ctDNA)	With alterations in the CEBPA, FGFR4, MET, or KMT2B genes in ctDNA (84).	
C-reaction protein (CRP)	Elevated CRP levels that CRP ≥ 8.2 mg/liter (*P* = 0.024) (85).	
Extracellular vesicle-derived proteins	Low levels of EV-ICOS and EV-IDO1 (86).	Highly expressed EV-ICOS and EV-IDO1 (86).

### 5.1 Blood counts and biochemical indicators

Circulating blood counts, such as neutrophil-lymphocyte ratio (NLR), platelet lymphocyte ratio (PLR), absolute eosinophilia count (AEC), and absolute basophil count (ABC) often objectively foresee the onset time of irAEs. Lower NLR (< 3) and PLR (< 180) were noticed in the irAE group, whereas higher AEC (> 240/μl) at baseline were related with endocrine irAEs (79).

A prospective follow-up of irAEs involving 1,187 cancer patients using anti–PD-1/L1 surprisingly identified factors associated with poor prognosis in solid cancer patients, such as elevated NLR and performance status (PS) ≥ 2 predicted very severe irAE and fatal irAE, and albumin levels < 35 g/liter predicted fatal irAE (80). Although the highest mortality rates have been reported for respiratory, cardiovascular, and nephrotoxicity, rather than gastrointestinal toxicity, it is worthwhile to alert clinicians to make early decisions so as to avoid serious gastrointestinal adverse events that affect patient quality of life and prognosis.

### 5.2 Genetic susceptibility

Genetic background of patients receiving ICI probably functions in irAE susceptibility.

It is clarified that the human leukocyte antigen (HLA) complex located at chromosome 6p21.3 is highly associated with the development of IBD, for example, HLA-DRB1*03:01 is the most relevant risk allele (87). However, it is unclear whether genetic HLA-mediated susceptibility to autoimmune disease contributes to the development of irAEs. Surprisingly, after analyzing the HLA alleles of nine patients who developed colitis after ICI treatment, Ali et al. found a nominally significant association between HLA-DQB1*03:01 and colitis, in other words, carriers of which were susceptible to colitis during immunotherapy (81). Owing to the very limited sample size, no insight into HLA-associated disease dynamics is possible, however, and it is essential to recruit a larger cohort for a more extensive genetic investigation.

The lymphocyte cytosolic protein 1 (LCP1) is involved in T-cell activation and the adenosine diphosphate dependent glucokinase (ADPGK) mediates metabolic shifts during T-cell activation. Combining real-world data with multi-omics data, Jing and coworkers developed a bivariate regression model for LCP1 and ADPGK, and validated it in a cohort of cancer patients receiving anti–PD-1/PD-L1 inhibitors, revealing the highest correlation between these two values and the presence of irAEs.

LCP1 (*P*  = 0.008) and ADPGK (*P* = 0.010) were higher in patients with irAEs than in those without irAEs, which suggests LCP1 and ADPGK may serve as biomarkers in an independent patient-level cohort (17).

Another retrospective study discovered that mutations in the oncogenic gene BRAF induced a lower probability of irAEs (*p* = 0.0180), while mutations in the NRAS gene may be positively associated with the development of irAEs (*p* = 0.2844). However, given the lack of prospectively collected mutational status, the association of oncogenic drivers with irAE occurrence requires further exploration to unravel (82).

A growing body of research has recently demonstrated the benefits of Vitamin D in tumor management by inhibiting tumor angiogenesis and microenvironmental inflammation (88). With Luo et al. (83) genotyping of 13 single-nucleotide polymorphisms (SNPs) in the Vitamin D metabolic pathway demonstrated that patients with the *CYP24A1* rs6068816TT and rs2296241AA genotypes were more likely to benefit from anti–PD-1 drugs. Three SNPs (*CYP24A1* rs2762934, *CYP27B1* rs10877012, and *SEC23A* rs8018720) reduced the risk of irAE in patients. However, although plasma 25(OH)D levels were associated with good disease control rates in patients, there was no statistical correlation with the risk of irAE.

### 5.3 Dynamic circulating tumor DNA

With assessment of 46 patients with advanced gastric cancer (AGC) who received anti–PD-1 immunotherapy and next-generation sequencing (NGS) testing, Jin and his colleagues observed the median PFS of 7.4 months *versus* 4.9 months for patients with undetectable and detectable post-treatment ctDNA (84). In other words, lower ctDNA was associated with a higher response to immunotherapy.

Common adverse reactions in enrolled patients were endocrine manifestations and hepatotoxicity, which were more likely to occur in patients with alterations in the CEBPA, FGFR4, MET, or KMT2B genes in ctDNA (84). So dynamic ctDNA is hopeful to be a biomarker for predicting irAEs, and we expect a larger cohort to conduct further exploration of its potential role in gastrointestinal toxicity.

### 5.4 C-reaction protein

C-reaction protein (CRP) is an acute phase protein synthesized by the liver and is markedly elevated in the early stages of inflammation or infection. Baseline CRP values cannot reliably predict irAE, but elevated CRP levels seem to correlate with irAE, with researchers noting that CRP ≥ 8.2 mg/liter (*P* = 0.024) were independent risk factors (85). However, because CRP is inherently elevated in patients with malignancy and the use of hormones can have an impact on its measurement, it is necessary to pay attention to whether the reduction in CRP correlates with improvements in other factors when assessing the recovery of patients with irAEs.

### 5.5 Extracellular vesicle-derived proteins

Extracellular vesicles (EVs) are membrane-bound nanovesicles that are secreted and released by almost all cells carrying a variety of bioactive molecules such as DNA, mRNA and proteins. Not only are they involved in signal transduction in intercellular settings, but a growing body of evidence clarifies that they are also likely to be non-invasive markers for cancer diagnosis and treatment (89). According to recent evidence, inducible co-stimulator (ICOS), served as a co-stimulatory receptor for T-cell enhancement, plays a dual role in different tumor types. As it enhances the production of CD8+ tissue-resident memory (Trm) T cells to strengthen the anti-tumor response, while promoting tumor progression by depressing the function of Tregs (90, 91). Indoleamine 2, 3-dioxygenase 1 (EV-IDO1) is a rate-limiting metabolic enzyme highly expressed in a variety of human cancers, although its functional effects in different cancer types are complex and remain to be investigated in relation to prognosis (92). Increased expression of IDO1 in tumors is accompanied by an increase in other immune checkpoints such as PD-1 (92). Hence, treating PD-1 inhibitors together with IDO1-targeted blockers may have a synergistic effect on immunotherapy, but whether there is a corresponding risk of additional toxicity is still unknown.

EV-ICOS and EV-IDO1 were extracted from the peripheral blood of patients with gastric cancer. Jiang et al. (86) reported firstly that these two EV-proteins were highly expressed in patients without irAEs. In addition, for the time interval between the initiation of ICI therapy and the development of irAEs, patients with high-baseline ICOS and IDO1 were slightly longer than those with low baseline ICOS and IDO1 expression. That is, patients carrying low levels of EV-ICOS or EV-IDO1 had a higher risk of irAEs and a shorter interval. Interestingly, these two proteins neither affected the long-term immunotherapy outcome that no changes in PFS and OS were seen, but the levels of the circulating tumor marker CA72.4 were highly positively correlated with them, meaning that they may affect short-term efficacy. Furthermore, EV-derived ICOS/IDO1 was not sufficient to predict organ-specific irAE.

## 6 Risk factors: individualized toxicity monitoring

Regarding the correlation between age and irAEs, scholars have conducted studies. Treating tumor-bearing mice with PD-1 inhibitors, Tsukamoto et al. noted significant aberration in biochemical indicators of organ dysfunction in aged mice, such as elevated alanine aminotransferase (ALT), triglyceride, amylase, urea nitrogen, ureic acid, surfactant protein D (SP-D), and abnormal lymphocytic accumulation, while juvenile mice unexpectedly showed no obvious irAEs-Like multi-organ toxicity (32). To study the response of elderly patients to treatment with PD-1 inhibitors, the group of 82 patients aged 65–79 years was defined as group A and the group of 62 patients aged 80–100 years was defined as group B. The investigators noted a trend toward higher ORR in group B compared with group A (73.9% *vs.* 62.3%) and a significantly higher CR rate in group B than in group A (47.9% *vs.* 20%), meaning that older melanoma patients over 80 years of age benefited the most. A weakened immune system with age may result in a better response to anti–PD-1 therapy. There was no significant difference in PFS or OS between the groups, probably because a considerable number of elderly patients were prone to die from complications other than melanoma progression such as ischemic heart disease. However, toxicity occurred significantly earlier in group B, that is, patients over 80 years of age, and may be related to more rapid kinetics of immune activation (93). Therefore, aging accelerates the disruption of immune tolerance, so that give rise to earlier onset of irAEs.

About the impact of gender, a prospective study of ipilimumab for melanoma clarified that women are at a higher risk of serious toxicity relative to men (94). In contrast, men who used PD-1 drugs and long-term smokers were at higher risk (79). Contradictory to findings mentioned above, in patients with cancer undergoing PD-1 blockade, neither age nor gender was noticed to influence the development of irAE, but high BMI (BMI > 25.0) made greater odds of irAE (82). Moreover, patients with underlying diseases such as hypertension, coronary heart disease, and chronic kidney disease have an elevated organ-specific irAE risk (79). Primary tumor histology also has an influence that distinct tumor types have histologically specific irAE patterns when treated with PD-1 suppressors, perhaps attributed to diverse TMEs and immune infiltration (81), with research identifying a higher risk of small bowel colitis in melanoma compared with NSCLC and renal cell carcinoma (95).

That irAEs attribute to dysregulation of immune system is resemble autoimmune diseases like IBD (23). Several studies have indicated that preexisting autoimmune disease increases the risk of irAE, probably because of enrichment of activated CD4^+^ T_M_ cells (15, 79). Not only that, Kayashima et al. noticed irAEs could lead to the follow-up progression of IBD (96). An elderly male with a previous history of left-sided involvement in ulcerative colitis was treated with anti–PD-1 drugs after the appearance of multiple organ metastases from kidney cancer, and subsequently experienced immune-mediated colitis with a primary site in the right transverse colon. Upon administration of prednisolone and multiple IFX to the patient, the condition was poorly controlled or even newly worsened, with inflammation extending to the rectum and sigmoid colon, which implies a realization of a shift in pathogenesis from irAE to ulcerative colitis recurrence (96). Whether to treat such patients with ICIs remains controversial, out of concern for symptom deterioration. In any case, frequent colonoscopies may be valuable for the management of such patients.

Early identification of high-risk patients facilitates diagnosis and timely treatment, although comprehensive predictive models are currently inaccessible (54).

## 7 The relationship between efficacy and safety

Does the development of irAEs affect the antitumor immune effect of ICI? How exactly does irAEs correlate with the survival outcomes and prognosis of anticancer process? In a large retrospective study investigating patients with various cancers treated with anti–PD-1 drugs, there were clinical benefits identified as remarkably higher objective response rate (ORR) (30.4% *vs.* 12.7%), notably prolonged median progression-free survival (PFS) (17.6 months *vs.* 3.0 months) and overall survival (OS) (48.7 months *vs.* 10.7 months) in patients with irAEs than in those without irAEs (97). Another meta-analysis including 4971 individuals showed irAEs, particularly low-grade irAEs, can be acted as predictive factors of a better ICI efficacy and prognosis, regardless of disease site (98). Relatively speaking, irAEs above grade 3 resulted in better ORR, but worse OS (99). Giving nivolumab to patients with AGC, Masuda et al. revealed the absence of irAEs indicated poor prognosis by multivariate analysis (100). Kono et al. also endorsed this view, contending a strongly positive association between irAEs and clinical benefit such as a longer OS in nivolumab-treated patients with AGC (101). Likewise, Jiang et al. noticed the occurrence of irAEs corresponds to a better disease control rate of immunotherapy in gastric cancer patients (86). Even, patients with multiple irAEs had a trend toward improved PFS and OS, as compared with patients with only a single irAE (82). Speaking from the studies listed above, irAEs mirror an early and timely immune activation against the tumor that should be considered as a surrogate marker for a positive response to ICI therapy (102). It is uncertain whether interrupted ICI therapy will limit long-term benefit on survival (103).

## 8 Rechallenge after irAEs

Scholars increasingly recognize that both the antitumor activity of ICIs and the accompanying adverse effects can be attributed to an overreaction of the immune system. There are a lot of debates if immunotherapy can be restarted after the onset of irAE.

In a large multicenter retrospective study, for the purpose of assessing the safety of ICI resumption after irAE, the researchers focused on patients who discontinued ICI therapy for episodes of immune-mediated diarrhea and colitis and resumed ICI therapy due to cancer progression or favorable response for continued maintenance therapy, it was detected that up to one-third of patients suffered colitis recurrence and must permanently cease ICI (104). But patients who used anti–PD-1/L1 drugs before the initial colitis episode had a higher risk of recurrent colitis; those who used anti–CTLA-4 at the time of ICI readministration had a higher risk of recurrence than those who used anti–PD-1/L1 and had a shorter time interval, which may be attributed to anti–CTLA-4 induced stronger Th17 memory in colitis than anti-PD/1, although no difference in severity was noted by Abu-Sbeih et al. (37, 104). Interestingly, compared with pembrolizumab, nivolumab appears to have a higher mean incidence of grade 3 or higher adverse events with an unknown mechanism (9).

Patients were closely monitored and it was seemingly safe to restart ICI therapy, as demonstrated by the fact that 61.1% of patients who discontinued ICI therapy owing to grade ≥ 2 irAEs did not experienced a recurrent grade ≥ 2 irAEs after reintroduction of the same ICI therapy (105). By analyzing case safety reports from the World Health Organization database VigiBase, Dolladille, and fellow revealed a 28.8% recurrent rate of the same irAE in patients retreated with the same ICI after an irAE. In a readministration, colitis was associated with a higher recurrence rate compared with other events (106). Despite the size, this cohort study did not assess differences in the severity of the two irAEs. According to Simonaggio et al., a second identical or different irAE of patients who were rechallenged with the same anti–PD-1/L1 agent were in a similar degree of severity to the first, but the interval between recurrent events was significantly shorter than the initial one (107). Concerning the observation of curative effect, the ORR and DCR after rechallenge were 43.1% and 71.9%, respectively, with no significant difference in comparison with the initial ICI treatment (108).

The guidelines have the following recommendations. Patients with G1 irAE generally continue ICI therapy but are monitored closely to avoid any symptom deterioration. G2 irAE patients should temporarily discontinue treatment and subsequently restart if there is a hormone reduction (e.g., prednisone ≤ 10 mg/day) and symptoms return to G1 or even disappear completely. Provided that the subjective desire of G3 irAE patients is fully considered, clinicians should evaluate whether the patient will gain benefit from resumed therapy without undue deliberation about the presumed risk of toxicity to influence decision making. To minimize the risk of recurrence of irAEs, the ASCO Guideline suggests that mucosal healing in repeated endoscopies and/or fecal calprotectin levels ≤ 116μg/g can be considered as indicators of the time to resume ICI therapy (64). Moreover, anti–PD-1/L1 drug therapy should be the preferred option, regardless of the initial regimen. G4 patients generally recommends permanent discontinuation of ICIs. However, patients with a history of autoimmune disease were not included in any of these studies, and potentially increased risk was not taken into account. In general, the decision to rechallenge is actually based on the potential risk-reward ratio, further large-scale prospective studies are unquestionably required to more thoroughly validate.

## 9 Rare cases

In the past few years, the rare gastrointestinal toxicity caused by PD-1 inhibitors has gained attention. Despite its rarity, it should be emphasized because delays in treatment may lead to a poor prognosis or even death.

### 9.1 Upper gastrointestinal inflammation

Mild upper gastrointestinal inflammation can be effectively managed with proton pump inhibitors, but deep ulcers usually require immunosuppressive therapy. After anti–PD-1 therapy, Collins et al. observed that some patients suffering from immune-associated colitis developed upper gastrointestinal inflammation in the form of CD8^+^ lymphocyte infiltration, manifested by dysphagia, nausea and vomiting, and even progression to necrotic gastritis. Up to 75% of them responded favorably to corticosteroids, so the application of corticosteroids seems necessary and effective in the case of severe toxicity (109).

### 9.2 Bowel perforation

Cho et al. presented a case of an esophageal cancer patient who underwent a second dose of nivolumab with complaints of abdominal pain and diarrhea. After the ineffectiveness of corticosteroids and antibiotics, a diagnostic laparoscopy revealed a perforated cecum with severe abdominal wall adhesions, severe inflammation and multiple ulcerations in the sigmoid colon, so that the patient was given an ileostomy ultimately until recovery from colitis (110).

A case is reported by Celli et al. of a melanoma patient treated with pembrolizumab who developed diffuse colitis that responded poorly to high-dose corticosteroids and IFX and even progressed rapidly to a fulminant colitis with multifocal ulcers and perforation, culminating in an emergent bowel resection (111).

In general, continuation of ICI therapy is not taken into consideration after the development of severe complications. However, Beck et al. reported a case of exceptional management. After pembrolizumab, a patient with lung cancer suffered from immune-mediated enterocolitis that progressed to small bowel perforation and ended up with partial small bowel resection and creation of a primary anastomosis. Considering that pembrolizumab was tolerated fairly well, tumor burden was significantly attenuated, and satisfactory outcome to treatment, it was still decided to reactivate pembrolizumab therapy (95). Whether permanent discontinuation of anti–PD-1 drugs after perforation is necessary needs to be decided carefully after taking into account the individualized therapy situation.

### 9.3 Intestinal obstruction

A case of colitis and secondary inflammatory intestinal obstruction in a liver cancer patient with sintilimab was recently reported. Tan and coworkers pointed out that surgery, a traditional means of relieving obstruction, should not be undertaken in order to avoid aggravating intestinal damage and postoperative complications (112). Furthermore, gastrointestinal decompression and parenteral nutrition in combination with glucocorticoids and somatostatin, a type of hormone known to inhibit the secretion of digestive juices and suppress inflammation, should be administered early to patients with severe abdominal distention and decreased anal exhaust after diagnosis of colitis to accelerate remission (112).

### 9.4 Celiac disease

Celiac disease (CeD) is a relatively rare form of irAE whose pathogenesis may be related to gluten-mediated activation of intestinal CD4^+^ T cells in the lamina propria (113). It is clinically identical to both duodenitis and colitis, with the exception that it usually does not require immunosuppressive therapy, so that early diagnosis by the presence of anti-tissue transglutaminase immunoglobulin (tTG-IgA) is crucial (114).

A recent case of an elderly male patient with melanoma receiving combination nivolumab and ipilimumab, presented with abdominal pain, diarrhea and generalized edema, positive serology for tTG-IgA, endoscopy suggestive of duodenal mucosal atrophy with duodenal biopsies confirming fulminant celiac disease. This patient improved with a gluten-free diet alone, defined as a decreasing trend in tTG-IgA, rather than immunosuppression (115).

Lacking appropriate biomarkers, clinicians warrant high vigilance for patients presenting with gastrointestinal distress after ICI therapy, preferably with early access to tTG-IgA titers. In case of non-response after empirical use of corticosteroids, a full gastrointestinal endoscopy with biopsy should be performed to clarify the etiology and prevent the emergence of fulminant events.

### 9.5 Appendicitis

The clinical manifestations (e.g., abdominal pain and fever) and imaging manifestations (e.g., appendiceal dilatation and wall thickening) of appendicitis after PD-1/PD-L1 inhibitors are similar to those of conventional appendicitis, so the current management strategies are largely similar (116). Despite the complexity of cancer patients, appendectomy remains the mainstay of treatment. Distinction between ICI-mediated colitis and appendicitis is of concern and is critical for follow-up. Diarrhea often occurs in cases of colitis. Moreover, a considerable proportion of colitis events will be early treated with hormones in the course of the disease, while antibiotics are not used because of negative effect on long-term survival (117); whereas in this case, appendicitis was managed without hormones or immunosuppression, instead, antibiotics were often used. Additionally, microbiota analysis has led to an improved understanding of colitis, and FMT has been shown to be effective in ICI-mediated colitis. Perhaps future studies with larger sample sizes can reveal that the microbiome characteristics of appendicitis patients to allow FMT to be equally beneficial for appendicitis patients. Improving the understanding of this adverse event is a critical unmet need to determine whether to continue ICI treatment after an appendicitis episode.

## 10 Conclusion

With the widespread application of PD-1 inhibitors, it brings satisfactory efficacy but also affects the patients’ life quality due to unpredictable irAEs. How targeted eliminate the adverse effects without compromising the antitumor activity of immunotherapy has become a continuously explored topic by scholars. We expect that a more comprehensive summary of the gastrointestinal adverse effects caused by PD-1 will lay the theoretical foundation for the improvement of relevant management measures in the future.

## Author contributions

YYC drafted the manuscript and generated the figure. FL and JL performed the background research. YDC, MX and SL were responsible for the documentation. LZ conceived of and designed the study as well as revised and edited the manuscript. All authors contributed to the article and approved the submitted version.
